# Prediction of conduction disturbances in patients undergoing transcatheter aortic valve replacement

**DOI:** 10.1007/s00392-023-02160-0

**Published:** 2023-01-21

**Authors:** Valérie Pavlicek, Felix Mahfoud, Katharina Bubel, Peter Fries, Sebastian Ewen, Michael Böhm, Bruno Scheller, Christian Ukena

**Affiliations:** 1 Klinik Für Innere Medizin III, Kardiologie, Angiologie Und Internistische Intensivmedizin, Universitätsklinikum Des Saarlandes, Saarland University, Kirrberger Str, 66421 Homburg/Saar, Germany; 2 Klinik Für Diagnostische Und Interventionelle Radiologie, Universitätsklinikum Des Saarlandes, Saarland University, Homburg/Saar, Germany; 3 Cape Heart Institute, Faculty of Health Sciences, University of Cape Town, Cape Town, South Africa

**Keywords:** Transcatheter valve replacement, Conduction disturbances

## Abstract

**Aim:**

Transcatheter aortic valve replacement (TAVR) can cause intraventricular conduction disturbances (ICA), particularly left bundle branch block (BBB) and high-degree atrioventricular block (HAVB). The aim of this study was to investigate clinical, anatomical, procedural, and electrophysiological parameters predicting ICA after TAVR.

**Methods:**

Patients with severe aortic stenosis (*n* = 203) without pacing devices undergoing TAVR with a self-expanding (*n* = 103) or balloon-expanding (*n* = 100) valve were enrolled. Clinical and anatomical parameters, such as length of the membranous septum (MS) and implantation depth, were assessed. His-ventricular interval (HVi) before and after implantation was determined. 12-lead-electrocardiograms (ECG) before, during and after 3 and 30 days after TAVR were analyzed for detection of any ICA.

**Results:**

Among 203 consecutive patients (aortic valve area 0.78 ± 0.18 cm^2^, age 80 ± 6 years, 54% male, left ventricular ejection fraction 52 ± 10%), TAVR led to a significant prolongation of infranodal conduction in all patients from 49 ± 10 ms to 59 ± 16 ms (*p* = 0.01). The HVi prolongation was independent of valve types, occurrence of HAVB or ICA. Fifteen patients (7%) developed HAVB requiring permanent pacemaker (PPM) implantation and 63 patients (31%) developed ICA within 30 days. Pre-existing BBB (OR 11.64; 95% CI 2.87–47.20; *p* = 0.001), new-onset left BBB (OR 15.72; 95% CI 3.05–81.03; *p* = 0.001), and diabetes mellitus (OR 3.88; 95% CI 1.30–15.99; *p* = 0.02) independently predicted HAVB requiring PPM. Neither pre-existing right BBB, a prolonged postHVi, increases in PR duration, any of the TAVR implantation procedural and anatomic nor echocardiographic characteristics were predictive for later HAVB.

**Conclusions:**

New-onset left BBB and diabetes mellitus independently predicted HAVB requiring PPM after TAVR and helped to identify patients at risk. Electrophysiologic study (EPS) of atrioventricular conduction was neither specific nor predictive of HAVB and can be skipped.

**Trial registration number:**

NCT04128384 (https://www.clinicaltrials.gov).

**Graphical abstract:**

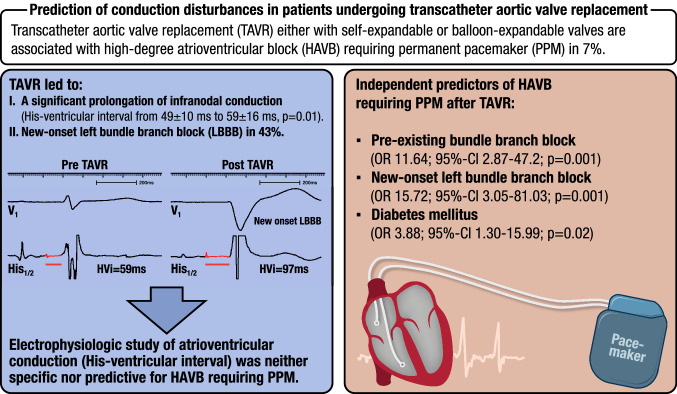

## Introduction

Transcatheter aortic valve replacement (TAVR) represents an established therapy for patients with severe aortic stenosis at intermediate to high surgical risk [1, 2]. Recent clinical trials suggest TAVR to be non-inferior or even superior in short- to mid-term outcomes in low-risk patients compared with surgical valve replacement (SAVR) [3]. Studies indicate that TAVR can cause intraventricular conduction abnormalities (ICA), particularly left bundle branch block (BBB) and high-degree atrioventricular block (HAVB) requiring permanent pacemaker (PPM) implantation [4, 5]. The development of symptomatic bradycardia results from mechanical stress to the left bundle branch and/or the atrioventricular node due to the deployed valve leading to HAVB [6]. Self-expandable valves have been associated with higher rates of PPM implantations than balloon-expandable prostheses. Right BBB at baseline independently predicted HAVB. Pre-existing left BBB did not [7]. Data on ICA, in particular on new-onset left BBB, and on progression to HAVB are scarce and conflicting. The present prospective study aimed to systemically investigate predictors of ICA after TAVR with self-expandable and balloon-expandable valves including an electrophysiologic study (EPS), especially of the atrioventricular conduction.

## Methods

### Study design

**Fig. 1:**
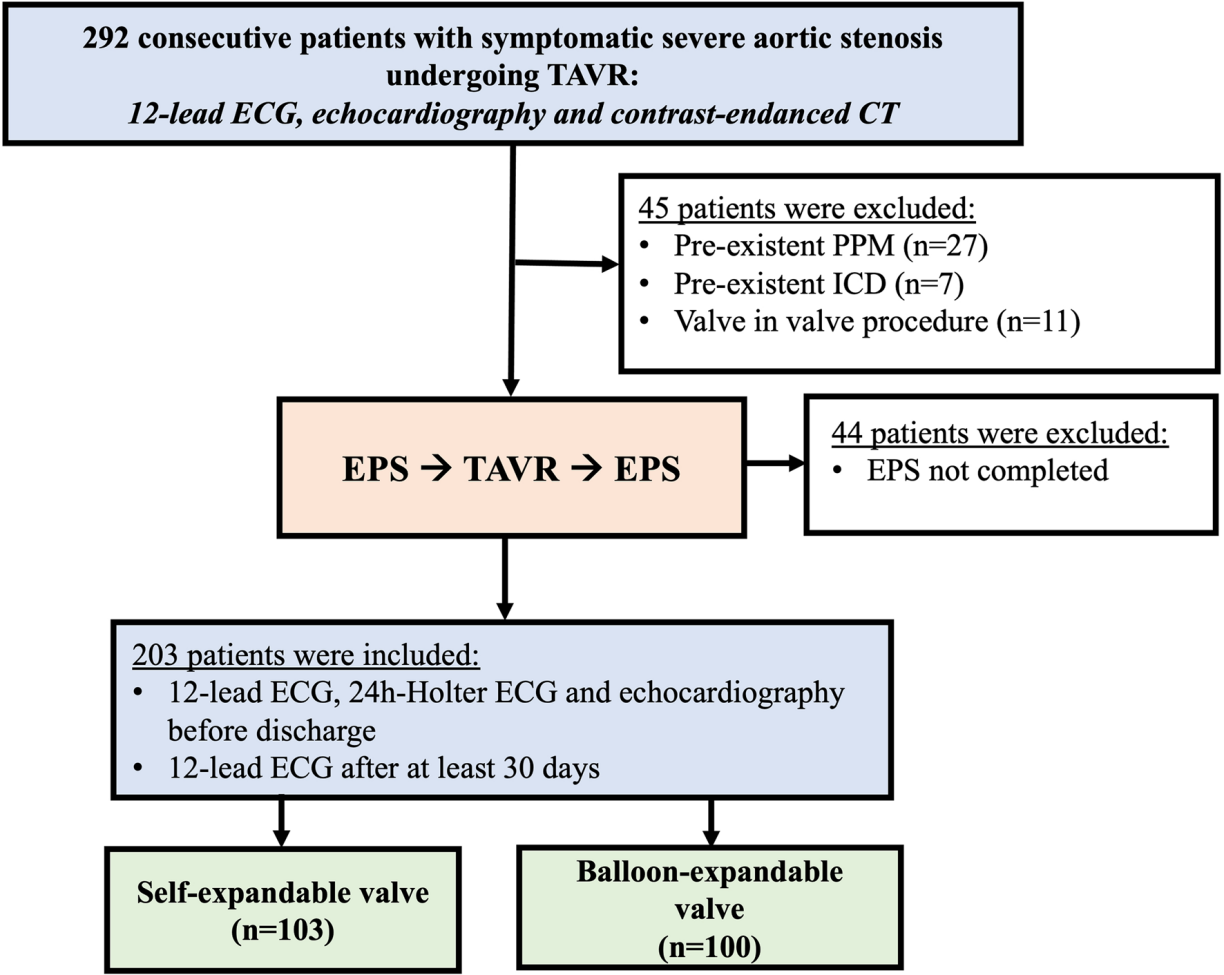
Study flow chart. *TAVR* transcatheter valve replacement, *ECG* electrocardiogram, *CT* computed tomography, *PPM* permanent pacemaker, *ICD* implanted cardioverter defibrillator, *EPS* electrophysiologic study

A total of 292 consecutive patients with severe aortic stenosis undergoing TAVR with a self-expandable valve (CoreValve EvolutR^®^, Medtronic Inc., Minneapolis, Minnesota, USA) or a balloon-expandable valve (SAPIEN 3^®^, Edwards Lifesciences Corporation, Irvine, California, USA) at Saarland University Center were screened. Inclusion criteria were the presence of symptomatic, degenerative aortic stenosis; an aortic valve area < 1 cm^2^ or an effective orifice area index < 0.6 cm^2^/m^2^; an aortic valve mean gradient > 40 mmHg or a jet velocity ≥ 4.0 m/s. The decision to perform TAVR was reached by consensus of the institutional heart team. Patients with pre-existing intracardiac devices (PPM (*n* = 27) and implanted cardioverter defibrillator (*n* = 7)), undergoing a valve in valve procedure (*n* = 11) and with not completed electrophysiologic study (EPS) (*n* = 44) were excluded. A total of 203 patients were finally included (Fig. 1).

As part of the pre-procedural work-up, all patients underwent 12-lead electrocardiograms (ECG), echocardiography, contrast-enhanced computed tomography angiography with ECG-synchronization for assessment of the anatomy and dimension of the aortic valve annulus, iliofemoral arteries, and the measurement of the length of the atrioventricular membranous septum, as described elsewhere [8]. The implantation depth was determined fluoroscopically in the implantation projection, the implantation depth was defined as the average distance from the native aortic annulus plane to the most proximal edge of the implanted valve (deepest level in the left ventricle) as previously described [8]. Medical history of all patients was taken on admission. Before discharge, a 12-lead ECG, 24-h Holter ECG and echocardiography were performed (Fig. 1). A transient left BBB was defined as the occurrence of new-onset left BBB that resolved before hospital discharge, and persistent left BBB as any new-onset left BBB that persisted after hospital discharge.

The study was approved by the local ethics committee based on ethical guidelines of the Declaration of Helsinki, and informed consent was obtained in all cases (NCT04128384).

### Transcatheter aortic valve implantation

The femoral artery and vein were punctured percutaneously, and the valve implanted according to the instruction for use. After the valve implantation, supravalvular angiography was performed to exclude paravalvular leakage. In case of relevant paravalvular leakage, balloon dilatation of the valve was performed. After removal of the delivery system, iliofemoral angiography was performed to exclude vascular complications. The femoral puncture site was closed using a hemostatic device (Perclose ProGlide^®^ Suture-Mediated Closure System, Abbott, Santa Clara, California, USA).

### Electrophysiological study (EPS)

A focused EPS was performed in all patients directly before and immediately after TAVR. A quadripolar standard diagnostic catheter (5F, Woven, Boston Scientific) was positioned at the His bundle to measure HVi and atrial-His interval (AHi), and thereafter maneuvered in the right ventricle apex for temporary pacing during valve deployment. After TAVR, the quadripolar diagnostic catheter was re-positioned at the His bundle for post TAVR measurements. In case of HAVB post TAVR, the catheter remained in the ventricular position for pacing.

### Electrocardiographic assessment

In all patients, resting 12-lead ECG (recording speed 50 mm/s; standardized calibration for 10 mm/mV) was recorded the day before, immediately after, after 3–5 days (before discharge) and at least 30 days post TAVR. All tracings were analyzed by two experienced cardiologists blinded to the time point of the recording and results of the EPS. Left BBB was defined as a QRS duration ≥ 120 ms with broad notched or slurred R wave in leads *I*, aVL, *V*_5_ and *V*_6_ and occasional RS pattern in *V*_5_ and *V*_6_ attributed to displaced transitions of QRS complex, absence of q waves in leads *I*, aVL, *V*_5_ and *V*_6_ (a narrow q wave can be present), R wave with slow growth in *V*_1_ to *V*_3_ with possible occurrence of QS, widened S waves with thickening and/or slots in *V*_1_ and *V*_2_, intrinsicoid deflection in *V*_5_ and *V*_6_ ≥ 55 ms, electrical axis between − 30° and + 60°; ST depression and asymmetrical T wave in opposition to medium-terminal delay.

### Follow-up and endpoints

After TAVR, patients were rhythm-monitored for at least 24 h at an intermediate care unit continuously. Afterward, we referred the patients to the normal care unit for an additional 3 to 5 days. Before discharge, a 24-h Holter monitoring was performed in every patient. At least 30 days after TAVR, patients´ follow-up included assessment of adverse events, physical status, and 12-lead-ECG. The primary endpoint was defined as the occurrence of HAVB (i.e., second-degree atrioventricular block Mobitz type 2 or complete atrioventricular block) requiring PPM implantation within 30 days after TAVR. A new-onset persistent left BBB and HAVB were defined as an intraventricular conduction abnormality (ICA) and the occurrence of ICA within 30 days after TAVR as secondary endpoint. All endpoints were adjudicated by two independent investigators (SE, DM).

### Statistical analysis

Data are presented as mean ± standard deviation (SD), median (interquartile range) or number (percentage) unless otherwise specified. Comparisons within groups were performed using the Pearson chi-square test for categorical variables and the Wilcoxon rank-sum test, the Kruskal–Wallis *H* test, or a paired t test for continuous variables where appropriate. A two-tailed *p*-value of < 0.05 was regarded as statistically significant. Regression analysis was performed to assess the association of parameters with the primary endpoint. After univariate screening, any candidate variable with a value of *p* < 0.05 as well as age and gender were forced to enter a multivariate model. All statistical analyses were performed with SPSS statistical software (version 21.0, SPSS Inc., Chicago, Illinois).

## Results

### Baseline characteristics

**Fig. 2:**
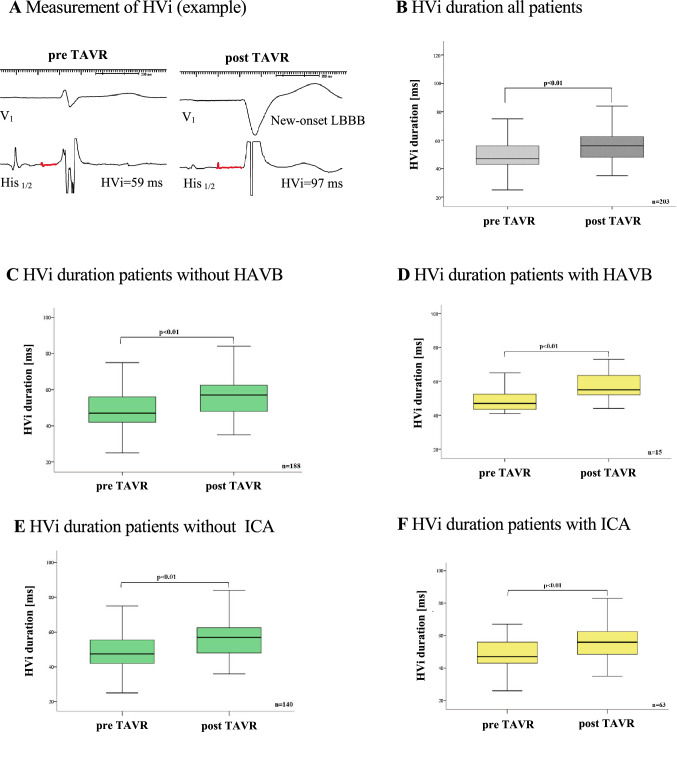
Duration of the His-ventricular interval (HVi) pre and post transcatheter valve replacement (TAVR). **A** Example for a measurement of HVi during electrophysiologic study. **B** HVi duration pre TAVR (49 ± 10 ms) and post TAVR (59 ± 16 ms) in all patients (*n* = 203, *p* < 0.01). **C** HVi duration pre TAVR (49 ± 10 ms) and post TAVR (58 ± 16 ms) in patients with no high-degree atrioventricular block (HAVB) requiring permanent pacemaker (PPM) (*p* < 0.01, *n* = 188). **D** HVi duration pre-TAVR (49 ± 7 ms) and post-TAVR (62 ± 17 ms) in patient with HAVR requiring PPM (*p* < 0.01, *n* = 15). **E** HVi duration pre-TAVR (49 ± 10 ms) and post-TAVR (58 ± 15 ms) in patients with no intraventricular conduction abnormality (ICA) (*p* < 0.01, *n* = 140). **F** HVi duration pre-TAVR (49 ± 10 ms) and post TAVR (58 ± 18 ms) in patients with ICA (*p* < 0.01, *n* = 63)

A total of 203 patients (age 80 ± 6 years, 54% male, left ventricular ejection fraction 52 ± 10%) with symptomatic severe aortic stenosis (aortic valve orifice area index 0.39 ± 0.10 cm^2^/m^2^, mean aortic valve gradient 45 ± 15 mmHg) were included. Baseline characteristics of the patients are shown in Table 1. A self-expandable valve was implanted in the first 103 patients, a balloon-expandable valve in the subsequent 100 patients. Besides the aortic valve orifice area (0.70 ± 0.19 cm^2^ vs. 0.76 ± 0.17 cm^2^, *p* = 0.01) and the implanted valve size, there were no significant differences regarding the baseline characteristics between the two valve types. The most frequently implanted self-expandable valve size was 29 mm, which was implanted in 53 patients (52%) followed by 26 mm in 30 (29%), 34 mm in 16 (16%) and 23 mm in 4 patients (4%). The most frequently implanted balloon-expandable valve size was 26 mm, which was implanted in 44 patients (44%) followed by 23 mm in 42 (42%), 29 mm in 13 (13%) and 20 mm in 1 patient (1%). Pre TAVR, the mean HVi duration was 49 ± 10 ms which significantly increased to 59 ± 16 ms post TAVR in all patients (*p* < 0.01) (Fig. 2a, b).

**Table 1:** Baseline characteristics

Characteristic	All (*n* = 203)	Self-expandable valve (*n* = 103)	Balloon-expandable valve (*n* = 100)	*P* value
Age [years], mean ± SD	80 ± 6	80 ± 7	81 ± 5	0.43
Male gender, *n* (%)	106 (52)	56 (54)	50 (50)	0.53
*Cardiovascular risk factors/comorbidities*				
Hypertension, * n* (%)	196 (9)	99 (96)	97 (97)	0.73
Diabetes mellitus, * n* (%)	74 (37)	37 (36)	37 (37)	0.87
Hyperliproteinemia, * n* (%)	150 (74)	72 (70)	78 (78)	0.19
Smoker, * n* (%)	42 (21)	20 (19)	22 (22)	0.65
Obesitas [BMI > 30 kg/m^2^], * n* (%)	45 (22)	19 (18)	26 (26)	0.20
Atrial fibrillation/atrial flutter, * n* (%)	65 (32)	30 (29)	35 (35)	0.37
Coronary artery disease, * n* (%)	120 (59)	56 (54)	64 (64)	0.16
Previous CABG, * n* (%)	20 (10)	9 (9)	11 (11)	0.59
Previous stroke/TIA, * n* (%)	30 (15)	16 (16)	14 (14)	0.76
Chronic kidney disease [eGFR < 60 ml/min/1.73m^2^], * n* (%)	96 (47)	46 (45)	50 (50)	0.45
Malignant disease/cancer, * n* (%)	45 (22)	19 (18)	26 (26)	0.20
COPD/Interstitial lung disease, * n* (%)	43 (21)	20 (19)	23 (23)	0.53
*Echocardiographic parameters*				
Left ventricular ejection fraction [%], mean ± SD	52 ± 10	52 ± 11	51 ± 10	0.86
*Aortic valve parameters*				
Aortic valve orifice area [cm^2^], mean ± SD	0.73 ± 0.18	0.70 ± 0.19	0.76 ± 0.17	**0.01**
Aortic valve effective orifice area index [cm^2^/m^2^], mean ± SD	0.39 ± 0.10	0.38 ± 0.10	0.40 ± 0.09	0.17
dpmax [mmHg], mean ± SD	71 ± 22	68 ± 20	74 ± 24	0.08
dpmean [mmHg], mean ± SD	45 ± 15	43 ± 13	47 ± 16	0.10
*Computed tomography parameters*				
Membranous septum length [mm], mean ± SD	8.5 ± 2.2	8.3 ± 2.3	8.7 ± 2.0	0.18
Calcification in basal septum, * n* (%)	51 (25)	20 (19)	31 (31)	0.06
**TAVR**				
*Valve size*				
20 mm, * n* (%)	1 (0.5)	0 (0)	1 (1)	< 0.01
23 mm, * n* (%)	46 (23)	4 (4)	42 (42)	< 0.01
26 mm, * n* (%)	74 (37)	30 (29)	44 (44)	< 0.01
29 mm, * n* (%)	66 (33)	53 (52)	13 (13)	< 0.01
34 mm, * n* (%)	16 (8)	16 (16)	0 (0)	< 0.01
Pre-dilatation, * n* (%)	8 (4)	2 (2)	6 (6)	0.14
Post-dilatation, * n* (%)	32 (16)	15 (15)	17 (17)	0.63
*Baseline 12-lead ECG*				
Sinus rhythm, * n* (%)	164 (81)	85 (83)	79 (79)	0.31
Atrial fibrillation / Atrial flutter, * n* (%)	37 (19)	16 (17)	21 (21)	0.31
Heart rate [bpm], mean ± SD	72 ± 16	74 ± 16	70 ± 15	0.09
PR interval duration [ms], mean ± SD	184 ± 45	180 ± 19	187 ± 51	0.33
AVB I, * n* (%)	46 (23)	124 (24)	22 (22)	0.83
QRS duration [ms], mean ± SD	100 ± 21	98 ± 19	102 ± 24	0.28
Bundle branch block, * n* (%)	49 (25)	27 (27)	22 (22)	0.48
LBBB, * n* (%)	26 (13)	14 (14)	12 (12)	0.66
RBBB, * n* (%)	6 (3)	3 (3)	3 (3)	0.66
Bifascicular block [RBBB + LAFB], * n* (%)	2 (1)	0 (0)	2 (2)	0.66
Incomplete LBBB, * n* (%)	6 (3)	3 (3)	3 (3)	0.66
Incomplete RBBB, * n* (%)	1(0.5)	1 (1)	0 (0)	0.66
LAFB	9 (5)	6 (6)	3 (3)	0.66
*Medication*				
Betablocker, * n* (%)	168 (85)	87 (85)	81 (81)	0.88
Amiodarone, * n* (%)	7 (4)	4 (4)	3 (3)	0.78
Digitalis, * n* (%)	6 (3)	3 (3)	3 (3)	0.92

Values are presented as mean ± standard deviation for continuous variables and as *n* (%) for categorical variables. *BMI* body mass index, *CABG* coronary artery bypass graft, *TIA* transient ischemic attack, *eGFR* estimated glomerular filtration rate, *COPD* chronic obstructive pulmonary disease, *TAVR* transcatheter aortic valve replacement, *AVB* atrioventricular block, *LBBB* left bundle branch block, *RBBB* right bundle branch block, *LAFB* left anterior fascicular block

### Occurrence of HAVB requiring PPM implantation (primary endpoint)

Overall, 15 patients (7%) developed a HAVB requiring PPM implantation within 30 days after TAVR. An immediate HAVB (< 24 h after TAVR) occurred in six patients (3%), a delayed HAVB (> 24 h and < 30 days after TAVR) in 9 patients (4%). A delayed HAVB occurred after a median of 7 days (2–28 days).

At baseline, there was a significantly higher rate of atrial fibrillation or flutter in patients developing a HAVB post TAVR (*p* = 0.03). Furthermore, patients developing a HAVB had a significantly longer QRS duration (99 ± 20 ms without HAVB (*n* = 188) vs. 113 ± 30 ms with HAVB (*n* = 15), *p* = 0.02) at baseline. Accordingly, more than a half of the patients with HAVB had a pre-existing BBB (41 (22%) patients (*n* = 188) without HAVB vs. 8 (53%) patients with HAVB (*n* = 15), *p* = 0.01). After TAVR, the QRS duration was correspondingly longer in patients reaching the primary endpoint (116 ± 29 ms in patients with without HAVB vs. 153 ± 29 ms in patients with HAVB, *p* < 0.001), and the increase in QRS duration was higher (8 (− 30;86) ms in patients without HAVB vs. 51 (8;82) ms in patients with HAVB, *p* = 0.01). A higher number of patients in the HAVB group had diabetes (64 (34%) patients without HAVB (*n* = 188) vs. 10 (67%) patients with HAVB (*n* = 15), *p* = 0.01) and were on antiarrhythmic medication with amiodarone (5 (3%) patients without HAVB (*n* = 188) vs. 2 (13%) patients with HAVB (*n* = 15), *p* = 0.03) (for more details, see Table 2).

**Table 2:** Characteristics of patients with HAVB requiring PPM implantation (primary endpoint) and with occurrence of ICA (secondary endpoint)

Characteristic	No HAVB (*n* = 188)	HAVB (*n* = 15)	*p*-value	No ICA (*n* = 140)	ICA (*n* = 63)	*p*-value
Age [years], mean ± SD	80 ± 6	82 ± 6	0.33	80 ± 6	80 ± 6	0.82
Male gender, * n* (%)	99 (53)	7 (47)	0.66	72 (51)	34 (54)	0.74
*Cardiovascular risk factors/comorbidities*						
Hypertension, * n* (%)	181 (96)	15 (100)	0.45	135 (96)	61 (97)	0.89
Diabetes mellitus, * n* (%)	64 (34)	10 (67)	**0.01**	47 (34)	27 (43)	0.20
Hyperliproteinemia, * n* (%)	139 (74)	11 (73)	0.96	103 (74)	47 (75)	0.88
Smoker, * n* (%)	36 (19)	6 (40)	0.06	26 (19)	16 (25)	0.27
Obesitas [BMI > 30 kg/m^2^], * n* (%)	39 (21)	6 (40)	0.08	27 (19)	18 (29)	0.14
Atrial fibrillation/Atrial flutter, * n* (%)	57 (30)	8 (53)	0.07	44 (31)	21 (33)	0.79
Coronary artery disease, * n* (%)	109 (58)	11 (73)	0.24	83 (59)	37 (59)	0.94
Previous CABG, * n* (%)	17 (9)	3 (20)	0.17	14(10)	6 (10)	0.92
Previous stroke/TIA, * n* (%)	28 (15)	2 (13)	0.87	20 (14)	10 (16)	0.77
Chronic kidney disease [eGFR < 60 ml/min/1.73m^2^], * n* (%)	86 (46)	10 (67)	0.12	61 (44)	24 (54)	0.20
Malignant disease/cancer, * n* (%)	43 (23)	2 (13)	0.39	32 (23)	13 (21)	0.72
COPD/interstitial lung disease, * n* (%)	42 (22)	1 (7)	0.15	30 (21)	13 (21)	0.90
*Echocardiographic parameters*						
Left ventricular ejection fraction [%], mean ± SD	52 ± 10	52 ± 15	0.87	52 ± 10	51 ± 12	0.74
*Aortic valve parameters*						
Aortic valve orifice area [cm^2^], mean ± SD	0.72 ± 0.18	0.85 ± 0.19	**0.01**	0.71 ± 0.18	0.77 ± 0.18	**0.03**
Aortic valve effective orifice area index [cm^2^/m^2^], mean ± SD	0.39 ± 0.10	0.42 ± 0.11	0.21	0.38 ± 0.09	0.41 ± 0.10	**0.04**
dpmax [mmHg], mean ± SD	71 ± 21	75 ± 35	0.64	72 ± 20	69 ± 26	0.50
dpmean [mmHg], mean ± SD	45 ± 14	48 ± 23	0.64	45 ± 15	44 ± 17	0.61
*Computed tomografy parameters*						
Membranous septum length [mm], mean ± SD	8.5 ± 2.2	8.4 ± 2.1	0.94	8.4 ± 2.3	8.1 ± 1.7	0.12
Calcification in basal septum, * n* (%)	48 (26)	3 (20)	0.63	32 (23)	19 (30)	0.24
**TAVR**						
*Valve type*						
Self-expandable valve, * n* (%)	97 (52)	6 (40)	0.39	70 (50)	33 (52)	0.75
Balloon-expandable valve, * n* (%)	91 (48)	9 (60)	0.39	70 (50)	30 (48)	0.75
*Valve size*						
20 mm, * n* (%)	1 (1)	0 (0)	0.61	1 (1)	0 (0)	0.21
23 mm, * n* (%)	44 (23)	2 (13)	0.61	37 (26)	9 (14)	0.21
26 mm, * n* (%)	67 (36)	7 (47)	0.61	52 (37)	22 (35)	0.21
29 mm, * n* (%)	60 (32)	6 (40)	0.61	40 (29)	26 (41)	0.21
34 mm, * n* (%)	16 (8)	0 (0)	0.61	10 (7)	6 (10)	0.21
Pre-dilatation, * n* (%)	7 (4)	1 (7)	0.58	5 (4)	3 (5)	0.69
Post-dilatation, * n* (%)	30 (16)	2 (13)	0.79	23 (16)	9 (14)	0.70
*Baseline 12-lead ECG*						
Sinus rhythm, * n* (%)	157 (83)	9 (60)	**0.03**	114 (81)	63 (82)	0.84
Atrial fibrillation / Atrial flutter, * n* (%)	31 (17)	6 (40)	**0.03**	26 (19)	11 (18)	0.84
Heart rate [bpm], mean ± SD	73 ± 16	67 ± 16	0.19	73 ± 16	70 ± 16	0.27
PR interval duration [ms], mean ± SD	182 ± 45	202 ± 41	0.20	184 ± 47	182 ± 42	0.72
AVB I, * n* (%)	42 (23)	4 (27)	0.73	30 (22)	16 (26)	0.55
**QRS duration [ms], mean ± SD**	99 ± 20	113 ± 30	**0.02**	101 ± 22	97 ± 19	0.27
**Bundle branch block, *** n* (**%)**	41 (22)	8 (53)	**0.01**	34 (25)	15 (25)	0.96
LBBB, * n* (%)	23 (12)	3 (20)	**0.03**	23 (17)	3 (5)	0.01
RBBB, *n* (%)	5 (3)	1 (7)	**0.03**	5 (3)	1 (2)	0.01
Bifascicular block (RBBB + LAFB), * n* (%)	1 (0.5)	1 (7)	**0.03**	1 (1)	1 (2)	0.01
Incomplete LBBB, * n* (%)	4 (2)	2 (13)	**0.03**	1 (1)	5 (8)	0.01
Incomplete RBBB, * n* (%)	1 (0.5)	0 (0)	**0.03**	1 (1)	0 (0)	0.01
LAFB, * n* (%)	8 (4)	1 (7)	**0.03**	4 (3)	5 (8)	0.01
*Medication*						
Betablocker, * n* (%)	154 (82)	14 (93)	0.34	116 (85)	52 (83)	0.80
Amiodarone, * n* (%)	5 (3)	2 (13)	**0.03**	4 (3)	3 (5)	0.50
Digitalis, * n* (%)	6 (3)	0 (0)	0.48	5 (4)	1 (2)	0.43
*Procedural features*						
Prosthesis/LVOT diameter, mean ± SD	1.3 ± 0.3	1.3 ± 0.1	0.78	1.3 ± 0.26	1.3 ± 0.32	0.80
Implantation depth [mm], mean ± SD	8.0 ± 3.9	8.4 ± 4.4	0.69	7.7 ± 3.9	8.6 ± 3.7	0.16
MS length [mm]–implantation depth [mm] = ∆MSID, median (IQR)	0.9 (− 13,6;13.3)	1.8 (− 8.2;8.2)	0.88	1.2 (− 13.6; 13.3)	− 0.3 (− 9.4;8.3)	**0.04**
HV interval						
preHVi, mean ± SD	48 ± 10	49 ± 7	0.86	49 ± 10	49 ± 10	0.84
postHVi [ms], mean ± SD	58 ± 16	62 ± 17	0.43	58 ± 15	59 ± 18	0.57
∆HVi, median (IQR)	4 (0;78)	7 (0;45)	0.41	5 (0;78)	4 (0;64)	0.42
AH interval						
preAH, mean ± SD	104 ± 31 (n = 115)	116 ± 41(n = 9)	0.11	105 ± 30	104 ± 37	0.92
postAH, mean ± SD	106 ± 31 (n = 108)	132 ± 74 (n = 8)	**0.04**	108 ± 32	108 ± 42	0.98
∆AH, median (IQR)	3 (− 47;56)	3 (− 30;100)	0.92	3 (− 47;56)	3 (− 37;100)	0.91
12-lead-ECG post-TAVR						
PR interval duration [ms], mean ± SD	193 ± 39	239 ± 52	**0.02**	192 ± 38	199 ± 44	0.38
∆PR interval (pre/post), median (IQR)	10 (− 70;100)	7 (− 12;86)	0.93	8 (− 70;100)	16 (− 60;90)	0.26
∆PR interval > 20 ms prolongation, * n* (%)	50 (27)	1 (7)	0.68	36 (26)	15 (24)	0.76
QRS duration [ms], mean ± SD	116 ± 29	153 ± 29	** < 0.01**	106 ± 24	148 ± 18	** < 0.01**
∆QRS (pre/post), median (IQR)	8 (− 30;86)	51 (8;82)	**0.01**	4 (− 30;58)	52 (4;86)	** < 0.01**
New-onset LBBB, * n* (%)	81 (43)	12 (80)	**0.01**	33 (24)	60 (95)	** < 0.01**
New-onset transient LBBB, * n* (%)	32 (17)	1 (7)	** < 0.01**	33 (24)	1 (2)	** < 0.01**
New-onset persistent LBBB, * n* (%)	49 (26)	11 (73)	** < 0.01**	0 (0)	59 (93)	** < 0.01**

Values are presented as mean ± standard deviation or median (interquartile range (IQR)) for continuous variables and as *n* (%) for categorical variables

*PPM* permanent pacemaker, *ICA* intraventricular conduction abnormality, *BMI* body mass index, *CABG* coronary artery bypass graft, *TIA* transient ischemic attack, *eGFR* estimated glomerular filtration rate, *COPD* chronic obstructive pulmonary disease, *TAVR* transcatheter aortic valve replacement, *AVB* atrioventricular block, *LBBB* left bundle branch block, *RBBB* right bundle branch block, *LAFB* left anterior fascicular block, *LVOT* left ventricular outflow tract, *MS* membranous septum, *HVi* his ventricular interval, *AH* atrial-His interval

All 15 patients with HAVB necessitating a PPM implantation had a left BBB: 3 patients (20%) a pre-existing left BBB, 11 patients (73%) a new-onset persistent left BBB, and 1 patient (7%) a new-onset transient left BBB (Table 2).

### Procedural features (self-expandable vs. balloon-expandable valve)

The prosthesis to left ventricular outflow tract diameter ratio (prosthesis/LVOT diameter) was 1.29 ± 0.28, the implantation depth 8.0 ± 3.9 mm, and the median difference between MS length and implantation depth (∆MSID) 0.9 (− 13;13.3) mm in all patients (Table 3). The membranous septum length did not differ between the two valve types (8.3 ± 2.3 mm self-expandable valve vs. 8.7 ± 2.0 balloon-expandable valve, *p* = 0.18) (Table 1). The self-expandable valves were implanted deeper (implantation depth 9.1 ± 4.6 mm self-expandable valve vs. 6.9 ± 2.4 mm balloon-expandable valve, *p* < 0.001) due to the construction of the valve and in bigger valve sizes also resulting in a higher prosthesis/LVOT diameter ratio (1.35 ± 0.33 self-expandable valve vs. 1.23 ± 0.20 balloon-expandable valve, *p* = 0.002), and a lower median ∆MSID (− 0.5 (− 13.6;13.4) self-expandable valve vs. 1.9 (− 7.8;9.7) balloon-expandable valve, *p* < 0.001) compared to balloon-expandable valves. Despite these observations, the occurrence of HAVB requiring PPM (6 (6%) patients (*n* = 103) self-expandable vs. 9 (9%) patients (*n* = 100) balloon-expandable valve, *p* = 0.39) and of ICA (33 (32%) patients (*n* = 103) self-expandable vs. 30 (30%) patients (*n* = 100) balloon-expandable valve, *p* = 0.75) did not differ significantly (Table 2).

**Table 3:** Procedural features (self-expandable vs. balloon-expandable valve)

Characteristic	All (*n* = 203)	Self-expandable valve (*n* = 103)	Balloon-expandable valve (*n* = 100)	*p*-value
***Procedural features***				
Prosthesis/LVOT diameter, mean ± SD	1.29 ± 0.28	**1.35 ± 0.33**	**1.23 ± 0.20**	**0.002**
Implantation depth [mm], mean ± SD	8.0 ± 3.9	**9.1 ± 4.6**	**6.9 ± 2.4**	** < 0.001**
MS length [mm]–implantation depth [mm] = ∆MSID, median (IQR)	0.9 (− 13.6;13.3)	− **0.5 (**− **13.6;13.3)**	**1.9 (**− **7.8;9.7)**	** < 0.001**
HAVB, * n* (%)	15 (7)	6 (6)	9 (9)	0.39
ICA, * n* (%)	63 (31)	33 (32)	30 (30)	0.75

Values are presented as mean ± standard deviation or median (interquartile range (IQR)) for continuous variables and as *n* (%) for categorical variables

*LVOT* left ventricular outflow tract, *MS* membranous septum, *IQR* interquartile range, *HAVB* high-degree atrioventricular block, *ICA* intraventricular conduction abnormality

### Predictors of HAVB

The regression analysis results are summarized in Table 4. Univariate regression analysis revealed that a pre-existing BBB, a new-onset left BBB, and diabetes mellitus were significantly associated with the occurrence of HAVB requiring PPM after TAVR. Neither pre-existing right BBB, a prolonged postHVi > 55 ms (70 ms), an increase in PR duration > 20 ms, any of the TAVR implantation procedural, and anatomic characteristics (pre-/postdilatation, implantation depth, MS length) nor echocardiographic characteristics were predictive for later HAVB. The parameters age, gender, pre-existent BBB, new-onset left BBB and diabetes mellitus entered the multivariate analysis. All three, pre-existing BBB (OR 11.64; 95% CI 2.87–47.20; *p* = 0.001), new-onset left BBB (OR 15.72; 95% CI 3.05–81.03; *p* = 0.001) and diabetes mellitus (OR 3.88; 95% CI 1.30–15.99; *p* = 0.02) proved to be independent predictors of development of HAVB requiring PPM implantation.

**Table 4:** Univariate and multivariate regression analysis

Parameter	Univariate analysis		Multivariate analysis	
	OR (95%-CI)	*p*-value	OR (95%-CI)	*p*-value
*HAVB within 30d post TAVR*				
Age > 81 years (median)	0.86 (0.29–2.52)	0.79	1.45 (0.46–4.97)	0.55
Male gender	0.78 (0.27–2.26)	0.66	0.79 (0.23–2.66)	0.70
**Pre-existing BBB**	**3.99 (1.37–11.65)**	**0.01**	**11.64 (2.87–47.20)**	**0.001**
Pre-existing RBBB	0.99 (0.98–1.02)	0.73		
Pre-existing LBBB	1.7 (0.45–6.50)	0.43		
**New-onset LBBB post TAVR**	**5.28 (1.44–19.34)**	**0.01**	**15.72 (3.05–81.03)**	**0.001**
AVB I pre TAVR	1.23 (0.37–4.06)	0.74		
Atrial fibrillation/Atrial flutter at baseline	2.63 (0.91–7.59)	0.07		
LVEF < 45% (first quartile)	1.91 (0.62–5.91)	0.26		
LVEDD > 55 mm (third quartile)	2.04 (0.66–6.33)	0.22		
dpmean > 55 mmHg (third quartile)	1.90 (0.61–5.87)	0.27		
postHVi > 55 ms	0.79 (0.27–2.26)	0.66		
postHVi > 70 ms	1.43 (0.38–5.39)	0.60		
∆PR > 20 ms post TAVR	0.62 (0.06–6.12)	0.68		
Pre-dilatation	1.84 (0.21–16.0)	0.58		
Post-dilatation	0.81 (0.17–3.78)	0.79		
Implantation depth > MS length	0.66 (0.22–2.02)	0.47		
Prosthesis/LVOT diameter > 1	0.25 (0.05–1.33)	0.11		
**Diabetes mellitus**	**3.88 (1.27–11.82)**	**0.02**	**4.56 (1.30–15.99)**	**0.02**
Amiodarone	5.48 (0–97-31.01)	0.06		
Calcification in basal septum	0.72 (0.20–2.68)	0.63		

Values are presented as odds ratio (95%-confidence interval (CI)). Besides age and gender, univariate values with a *p*-value < 0.05 entered multivariate analysis

*PPM* permanent pacemaker, *TAVR* transcatheter aortic valve replacement, *BBB* bundle branch block, *RBBB* right bundle branch block, *LBBB* left bundle branch block, *AVB* atrioventricular block, *HVi* His-ventricular interval

### Occurrence of ICA (new-onset persistent left BBB or HAVB requiring PPM implantation, secondary endpoint)

Overall, 63 patients (31%) developed an ICA within 30 days after TAVR (Table 2) with a correspondingly longer QRS duration (148 ± 18 ms patients with ICA vs. 106 ± 24 ms patients with no ICA, *p* < 0.001) and median QRS duration increase post TAVR (52 (4;86) ms patients with ICA vs. 4 (− 30;58) ms patients without ICA, *p* < 0.001). Interestingly, the difference between MS length and implantation depth (∆MSID) with significantly decreased in patients developing ICA after TAVR (− 0.3 (− 9.4; 8.3) patients with ICA vs. 1.2 (− 13.6; 13.3) patients without ICA, *p* = 0.04). As with the primary endpoint, there were significant differences in terms of the aortic valve orifice area (0.77 ± 0.18 cm^2^ with ICA vs. 0.71 ± 0.28 cm^2^ no ICA, *p* = 0.03) and the aortic valve effective orifice area index (0.41 ± 0.10 cm^2^/m^2^ with ICA vs. 0.38 ± 0.09 cm^2^/m^2^ no ICA, *p* = 0.04). A new-onset left BBB was observed in 93 (46%) patients and resolved in 33 (16%) cases before discharge. In 59 (29%) patients, the new-onset left BBB remained persistent. Transient left BBB (*n* = 33) resolved in 16 (48%) patients within 24 h after TAVR, in 8 (25%) patients within 2 days, 5 (15%) patients within 3 days, and 4 (12%) patients within 4 days after TAVR. Prolongation of the HVi, as described above, was not associated with the implanted valve type, the need for PPM implantation and the occurrence of ICA post TAVR (Fig. 2). The mean post-HVi did not differ between patients with or without new-onset left BBB (59 ± 17 ms vs. 58 ± 25 ms, *p* = 0.78).

## Discussion

Transcatheter AVR has been shown to reduce morbidity and mortality in patients with severe aortic stenosis but can cause intraventricular conduction abnormalities. The value of measuring the HVi before and after TAVR for prediction of future conduction disturbances was unknown. The main findings of our study are: (1) significant HVi prolongation post TAVR did not indicate a higher risk for progression rate to HAVB during 30 days post TAVR, (2) pre-existing BBB, a new-onset left BBB after TAVR, and diabetes mellitus were independent predictors for development of a HAVB requiring PPM after TAVR, (3) other known risk factors could not be verified as predictors for HAVB post TAVR in our study, and (4) there were no differences neither in the occurrence of HAVB requiring PPM nor occurrence of ICA between the self-expandable and balloon-expandable valves.

### HVi prolongation post TAVR did not indicate a higher risk for progression rate to HAVB

We report a significant prolongation of the HVi by 22% after TAVR using a self-expandable valve and by 19% using a balloon-expandable valve. His bundle recordings have been used for several decades for risk assessment of HAVB in patients with BBB and syncope [4, 9]. Peri-procedural persistent and post-procedural HAVB were shown as the only predictors of late HAVB, both of which were low in our study [10]. High-degree AVB are related to infra-Hisian disorders, but prognostic value of HVi before and after TAVR remains debated. López-Aguilera et al. observed a prolongation of HVi after self-expandable TAVR, comparable with our data [11]. Others investigated the value of an EPS in patients undergoing TAVR identifying a prolongation of HVi by more than 13 ms as the only independent predictor for HAVB (sensitivity 100%, specificity 84.4%) [12].

A prolonged HVi ≥ 70 ms, or HVi ≥ 100 ms after pharmacological stress or induction of HAVB by atrial pacing or by pharmacological stress, identifies a group at higher risk of developing HAVB [4]. In our study, the prolongation of the HVi ≥ 70 ms could not be identified as an independent predictor for the occurrence of HAVB. We found no statistical prognostic value of any HVi nor delta-change in HVi to predict HAVB. Badenco et al. also observed no correlation of His bundle recordings before, immediately after and some days after TAVR with the occurrence of HAVB [13]. Therefore, the EPS does not represent a valuable tool for the risk stratification of HAVB after TAVR.

### Pre-existent BBB, a new-onset left BBB after TAVR and diabetes mellitus were independent predictors for development of a HAVB requiring PPM after TAVR

A pre-existing BBB was associated with an almost 12-fold increased risk of HAVB. The available data about the impact of new-onset left BBB undergoing TAVR are scarce. It was demonstrated that new-onset left BBB was associated with a doubling of PPM rates after TAVR and all-cause mortality [14]. A systematic review and meta-analysis showed an association of new-onset left BBB with an increased risk of PPM implantation [15]. In our study, a new-onset left BBB was identified as an independent predictor for PPM implantation. Fifteen patients developed a HAVB necessitating a PPM within 30 days post TAVR all having a left BBB: three patients a pre-existent left BBB, 11 a new-onset persistent left BBB, and one patient a new-onset transient left BBB. Recently published guidelines recommend an extended monitoring period of up to 5 days in patients with dynamic progression of conduction abnormalities as new-onset BBB with dynamic prolongation of QRS and/or PR after TAVR [4]. Interestingly, the new-onset transient left BBB after TAVR resolved within four days in our patients, which confirms the need for the extended monitor period as the new-onset left BBB was identified as an independent predictor for HAVB. Compared to a recent meta-analysis describing an occurrence of new-onset left BBBin approximately 23% after TAVR [16], the rate of new-onset left BBB in 46% cases was high in our study. As in our study, first-degree atrioventricular block has not been shown conclusively to be an independent predictor for PPM implantation after TAVR [17]. We observed a 4.6-fold higher risk for HAVB in diabetic patients. Interestingly, diabetes mellitus was identified as a clinical predictor of PPM implantation after TAVR. In a recent meta-analysis, diabetes mellitus was associated with an OR of 1.08 (1.03–1.13) for pacemaker implantation after TAVR [18]. Therefore, diabetes mellitus as potential risk factor for HAVB after TAVR deserves further investigations for understanding the underlying mechanism and methods to reduce the risk.

### Other known risk factors could not be verified as predictors for HAVB post TAVR in our study

The largest meta-analysis evaluating the incidence of PPM implantation and clinical predictors after TAVR including 29 113 patients identified baseline conduction abnormalities as right BBB, left BBB and first-degree AVB, self-expandable valves, age, and implantation depth as the main independent predictors of PPM overall [16]. Most likely, the pre-existent right BBB could not be identified as an independent predictor in our study, as the rate of pre-existent right BBB with 6 patients (3%) was too low. An pre-existent left BBB is present in about 10 to 13% of the patients undergoing TAVR [19] consistent with 13% of the patients having a pre-existent left BBB in our study. But even in this case, the pre-existent left BBB could not be observed as an independent predictor for HAVB. Only three patients receiving a PPM post TAVR had a pre-existent left BBB. The anatomic proximity of the aortic anulus and the nodal-Hisian conduction system explain the occurrence of conduction disturbances after TAVR. The depth at which a valve prosthesis is implanted in the left ventricular outflow tract has been consistently associated with HAVB requiring PPM implantation for both self- and balloon-expandable valves [7]. In a recent expert consensus paper, a prosthesis to left ventricular outflow tract diameter ratio > 1 was mentioned as a risk predictor developing conduction disturbances related to a TAVR procedure [17]. In our study, the mean prosthesis/LVOT ratio was 1.29 ± 0.28 with a higher ratio in TAVR using self-expandable valve (1.35 ± 0.35) meaning that all patients were at higher risk as described above. However, the prosthesis/LVOT ratio was not an independent predictor of HAVB or ICA either in our study. Similarly, the length of the membranous septum was described as a predictor for PPM after TAVR especially the difference between membranous septum length and implantation depth (∆MSID) [8]. Similar to the prosthesis/LVOT ratio, we could not identify ∆MSID as independent predictor.

### There were no differences neither in the occurrence of HAVB requiring PPM nor occurrence of ICA between the self-expandable and balloon-expandable valves

A meta-analysis on 11,210 patients, documented a rate of HAVB or PPM implantations of 6% for balloon-expandable valves (SAPIEN S3^®^) and 28% for self-expandable valves (EvolutR^®^). The difference may be explained by the larger structure of self-expanding valves and a deeper implantation into the left ventricular tract, which may result in an increased risk of injury to the AV node and the left bundle branches [20]. Also in our study, the implantation depth of the self-expandable valves was deeper, the prosthesis to left ventricular outflow tract diameter ratio higher and the median ∆MSID lower. However, we could neither observe a higher rate of HAVB requiring PPM nor of ICA in TAVR using the self-expandable valve.

## Limitations

Our study may have some limitations. First, it is a monocenter prospective study with a limited number of patients undergoing TAVR. Second, the HAVB rate necessitating PPM implantation of 7% was relatively low. Forty-four screened patients could not be included due to incomplete EP study. Further, the EP study was performed during the index procedure after deploying the valve prosthesis, but the conduction after TAVR might change, especially within the first 24 h [17]. The follow-up period with 30 days after TAVR could only represent data on short- to intermediate-term outcome.

Of note, HAVB may occur immediately but also later involving inflammatory processes in addition to mechanical injury caused by valve expansion in the outflow tract [6]. In the present study, an immediate persistent HAVB (< 24 h after TAVR) occurred in 6 patients (3%) leading to an early PPM implantation. However, HAVB may regress as previously described [21]. As the numbers of HAVB after TAVR are too low in our study, no conclusion on the course and relevance of intermittent or persistent HAVB can be drawn.

## Conclusions

Transcatheter AVR either with self-expandable or balloon-expandable valves is associated with HAVB and with ICA. More specific studies on atrioventricular conduction electrophysiology neither add information nor are predictive for HAVB after TAVR. However, new-onset left BBB and diabetes mellitus independently predict HAVB after TAVR and help to identify patients at risk.

## Data Availability

Data will be made available upon reasonable request.

## Abbreviations

BBB: Bundle branch block
ECG: 12-Lead electrocardiogram
EPS: Electrophysiologic study
HAVB: High-degree atrioventricular block
HVi: His-ventricular interval
ICA: Intraventricular conduction disturbance
PPM: Permanent pacemaker
TAVR: Transcatheter aortic valve replacement
